# Genetic analysis of parathyroid and pancreatic tumors in a patient with multiple endocrine neoplasia type 1 using whole-exome sequencing

**DOI:** 10.1186/s12881-017-0465-9

**Published:** 2017-10-02

**Authors:** Bo-Young Kim, Mi-Hyun Park, Hae-Mi Woo, Hye-Yeong Jo, Ji Hoon Kim, Hyung Jin Choi, Soo Kyung Koo

**Affiliations:** 1 Division of Intractable Diseases, Center for Biomedical Sciences, Korea National Institute of Health, 187 Osongsaengmyeing2-ro, Cheongju-si, Chungcheongbuk-do 28159 South Korea; 2 Department of Anatomy, Department of Biomedical Science, Neuroscience Research Institute, Seoul National University College of Medicine, 28 Yongon-dong, Chongno-gu, Seoul, South Korea; 3 Department of Surgery, Eulji University Hospital, Daejeon, South Korea

**Keywords:** Multiple endocrine neoplasia type 1, Genetic analysis, Somatic mutation, Whole-exome sequencing, Clinical genomics, Case report

## Abstract

**Background:**

Multiple endocrine neoplasia type 1 (MEN1) syndrome is an autosomal dominant hereditary disorder characterized by the presence of endocrine tumors affecting the parathyroid, pancreas, and pituitary. A heterozygous germline inactivating mutation in the *MEN*1 gene (first hit) may be followed by somatic loss of the remaining normal copy or somatic mutations in the *MEN1* gene (second hit). Whole-exome sequencing has been successfully used to elucidate the mutations associated with the different types of tumors.

**Case presentation:**

We performed whole-exome sequencing (WES) on three parathyroid tumors, one pancreatic insulinoma, and a blood sample taken from the same patient with MEN1 to study tumor heterogeneity in MEN1 originating from different tumors. We identified a novel frame-shift deletion (c.1382_1383delAG, p.E461GfsX69) in the *MEN1* gene using WES, which was confirmed by Sanger sequencing. WES and the SNP array revealed somatic LOH on chromosome 11 in parathyroid tumors (left upper, left lower, and right upper parathyroid). However, we did not detect a somatic MEN1 gene mutation or LOH in the pancreatic insulinoma. WES revealed two somatic functional variants outside the MEN1 gene in the pancreatic insulinoma.

**Conclusions:**

This study revealed heterogeneity among tumors in the same patient with MEN1, suggesting that different tumor-specific tumorigenic mechanisms may contribute to the pathogenesis of MEN1 tumors. The present study supports the clinical applicability of the WES strategy to research on multiple tumor samples and blood.

**Electronic supplementary material:**

The online version of this article (10.1186/s12881-017-0465-9) contains supplementary material, which is available to authorized users.

## Background

Multiple endocrine neoplasia type 1 (MEN1) is an autosomal dominant inherited tumor syndrome characterized by the combined tumors of the parathyroid glands, pancreas, and pituitary gland [1]. Parathyroid tumors are the most common clinical manifestation, and the prevalence of hyperparathyroidism is >90% in patients with MEN1, whereas those of pancreatic and pituitary tumors are 40–70% and 30–60%, respectively [2]. MEN1 is caused by germline mutations in the MEN1 tumor suppressor gene linked to chromosomal locus 11q13 [3]. A heterozygous germline-inactivating mutation of the *MEN1* gene (first hit) may be followed by loss of the normal copy of this gene or a somatic inactivating mutation (second hit), leading to complete loss of function of the encoded protein menin [4, 5]. A *MEN1* gene mutation analysis has helped identify the cause of various sporadically occurring tumors of the same type that are observed in patients with MEN1 syndrome.

Identifying the genetic cause of the disease has become useful for diagnosing at-risk carriers affected by MEN1, and whole-exome sequencing (WES) approaches could help identify the causative genes [6]. Such an approach has been successfully used to elucidate mutations associated with multiple tumor types. Since next-generation sequencing (NGS) technology was introduced in 2008 [7], comprehensive genomic analyses have accelerated. With the power to detect novel variants from only a small number of individuals, NGS is proving invaluable for modern geneticists, boasting putative diagnostic rates of 21–25% for rare diseases of unknown etiology [8, 9]. Therefore, we pursued a WES analysis of parathyroid adenomas and a pancreatic insulinoma to study the tumor heterogeneity that may be contributing to different mechanisms of tumor-specific tumorigenesis.

## Case presentation

**Fig. 1:**
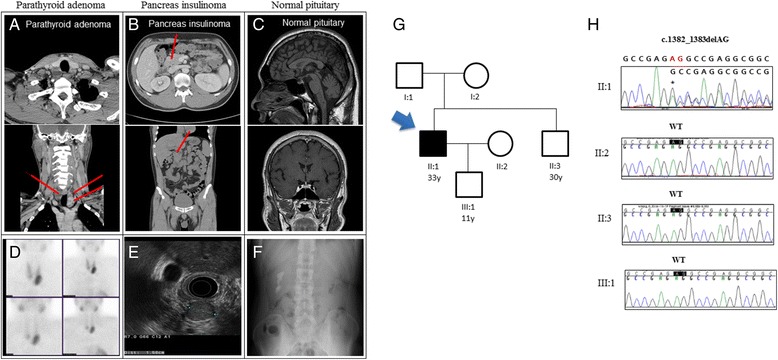
Case subject. **a** Neck computed tomography (CT) scan: Heterogeneous enhancing mass with cystic changes in both paratracheal (right 1.5 cm, left 2.2 cm) and parathyroid adenomas. **b** Abdominal CT: About a 1.6-cm well-defined enhancing mass at the head of the pancreas; R/O endocrine tumor. **c** Sellar magnetic resonance imaging: Normal pituitary. **d** Sestamibi parathyroid scan: Multiple parathyroid adenoma. **e** Endoscopic ultrasound and fine need aspiration: About a 1.6-cm-sized round hypoechoic homogenous mass at the pancreatic head. Immunohistochemistry: Ki-67 index <1%, P53: (−), synaptophysin: (+), neuroendocrine tumor. **f** KUB X-ray: Huge calyceal stones, Rt. kidney. **g** Pedigree of the patient with multiple endocrine neoplasia type1 (MEN1). The affected subject is indicated by the arrow. **h** Novel germline mutation in the MEN1 gene. Sanger sequence of MEN1 codons 462 in a wild-type subject and patient are shown and confirmed the c.1382_1383delAG mutation. Mutated residue is indicated by an asterisk, and the encoded amino acids are shown as single-letter code

The proband patient was admitted to hospital with a chief complaint of flank pain. The patient had been sweating for 5 years, and was nervous when fasting. The patient had had multiple episodes of kidney stones during the past 2 years. Table 1 presents the patient’s clinical information. The serum calcium level was 13.7 mg/dL (reference range, 8.2–10.8 mg/dL), and serum intact PTH was 209 pg/mL (reference range, 6–8 pg/mL), indicating primary hyperparathyroidism. The fasting serum glucose was 48 mg/dL (reference range, 70–110 mg/dL), and fasting serum insulin was 21.9 μU/mL, with a serum C-peptide level of 3.00 ng/mL (reference range, 1.06–3.53 ng/mL), indicating endogenous hyperinsulinemia. The anterior pituitary hormones were within the normal ranges. Sestamibi parathyroid, neck computed tomography (CT), and neck ultrasonography revealed multiple parathyroid adenomas. Abdominal CT revealed an approximately 1.6-cm well-defined enhancing mass at the head of the pancreas, suggesting an endocrine tumor. Brain sellar magnetic resonance imaging showed a normal pituitary. A subtotal parathyroidectomy and pancreaticoduodenectomy were performed. Postoperatively, all hormones and serum calcium returned to their normal ranges (Fig. 1).

**Table 1:** Clinical details of the patient with multiple endocrine neoplasia type1

Characteristic				Reference
Sex/Age (yr)	M/33			
Primary hyperparathyroidism (Parathyroid adenoma)				
Calcium	13.7	▲	mg/dL	8.2–10.8
Phosphorus	2.3	▼	mg/dL	2.5–5.5
PTH-intact	209	▲	pg/mL	8–6
PTH related peptide (PTHrP)	1.1			<1.1
Creatinine	2.00	▲	mg/dL	0.60–0.20
Insulinoma (enteropancreatic tumor)				
C-peptide (serum)	3.00		ng/mL	1.06–3.53
Glucose	48	▼	mg/dL	70–110
Insulin	21.9	▲	μIU/mL

### Whole-exome sequencing and validation with Sanger sequencing

Genomic DNA was isolated from matched tumor tissues and a constitutional blood sample using standard protocols included in the commercially available QIAGEN DNeasy Blood and Tissue kit (QIAGEN, Hilden, Germany). All specimens were quality control checked for purity using a NanoDrop spectrophotometer and Qubit Fluorometer (Thermo Fisher Scientific Inc., Waltham, MA, USA). Tumor and blood DNA were captured on the Agilent SureSelect DNA library construction (SureSelect V4 + UTR; Agilent Technologies, Palo Alto, CA, USA) and were sequenced on the Illumina Hiseq2000 instrument (Illumina, Inc., San Diego, CA, USA), as described by the manufacturer’s protocols for paired 101-bp reads. Image analysis and base calling was performed using Illumina pipeline ver. 2.3 with default parameters.

Reads were aligned to the human reference hg19 genome assembly using the Burrows–Wheeler Aligner (BWA; http://bio-bwa.sourceforge.net/), and duplicated read pairs were removed [10]. Genotypes for single nucleotide variants (SNVs) and short indels were called using the Genome Analysis Toolkit (GATK) [11] and Sequence Alignment Map tool (SAMtools, http://samtools.sourceforge.net/), respectively. Variants were annotated using ANNOVAR (http://www.openbioinformatics.org/annovar/). Allele frequencies for each variant were estimated based on phase 1 genotypes from the 1000 Genomics Project, the Korean control exome dataset, which includes the Korean Reference Genome (KRG database, *n* = 622). The effects of the identified variants were assessed using the Sorting Intolerant Form Tolerant (SIFT, http://sift.jcvi.org), and PolyPhen-2 (http://genetics.bwh.harvard.edu/pph2), automatic tools for predicting the possible impact of an amino acid substitution on the structure and function of a human protein [12, 13].

Sanger sequencing was used as an alternate platform to validate the identified variants in the four exome-sequenced tumor samples. Primer3 (http://frodo.wi.mit.edu/primer3/) was used to generate primers to amplify variants identified via exome sequencing. Amplicons from blood and tumor genomic DNA were analyzed using gel electrophoresis and were sequenced using an ABI 3730 genetic analyzer (Applied Biosystems, Foster City, CA) with forward and reverse primers.

### Genotyping and identification of loss of heterozygosity

Samples were genotyped with the Illumina Human Omni2.5–8 BeadChip according to the manufacturer’s protocols, and the genotypes were called using GenomeStudio software (Illumina) with standard cluster definitions. GenomeStudio provides raw data normalization, clustering, and genotype calling and calculates a GenCall score for each genotype, which is a measure of the accuracy of the genotype call. A 1-Mb sliding window across each chromosome was used to determine large regions with LOH in the tumor samples. The SNP array extracted both the log_2_ intensity ratio (LogR) and the B allele frequency (BAF) data. LogR measures the intensity of a sample relative to the reference intensity, and BAF measures the allele frequency at a particular SNP along the genome.

### Whole-exome sequence analysis

We performed WES on three parathyroid and one pancreatic tumor specimens as well as on a whole blood sample from the same patient diagnosed with MEN1. We sequenced the tumor and germline DNA isolated from whole blood to identify somatic changes in the tumors.

**Fig. 2:**
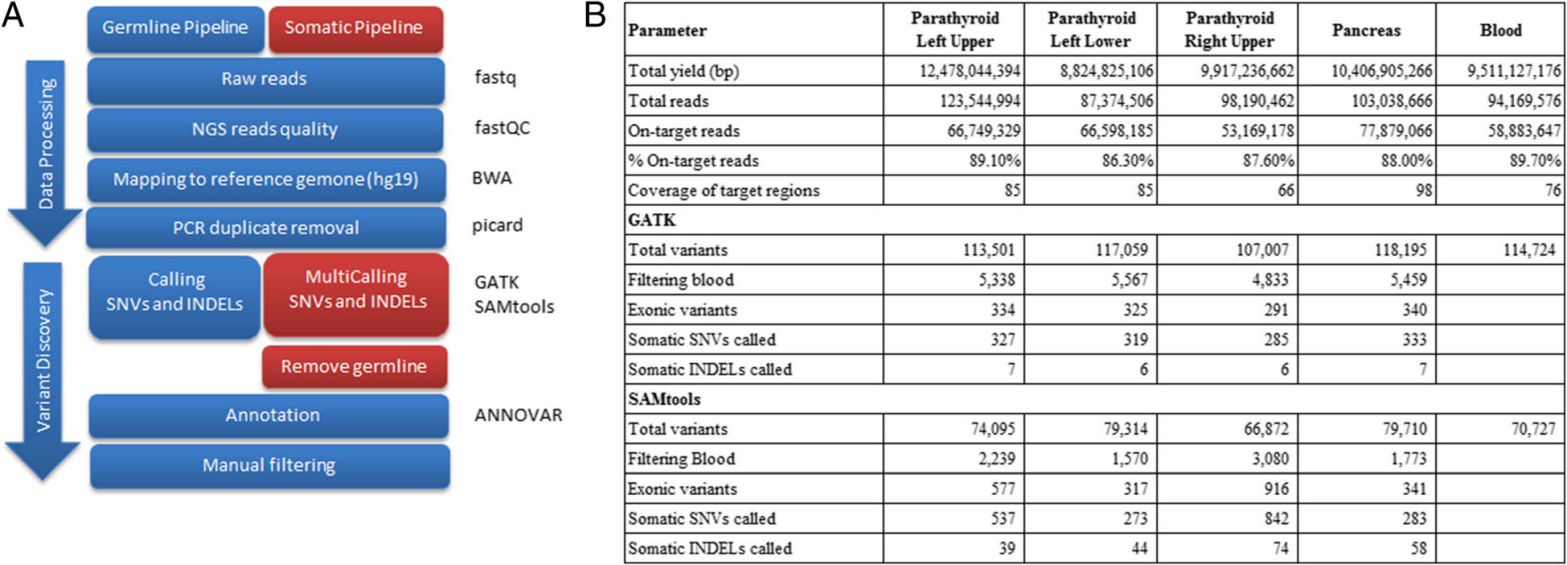
Whole-exome sequencing analytical pipelines. **a** Two workflows are present: Germline detection and somatic detection. Red highlights the difference between our germline pipeline and the somatic pipeline. **b** Summary exome sequencing statistics for all four tumor tissues and blood

We provided an analytical pipeline to detect germline or tumor-specific somatic variations in which each sample was considered independently. The genomic pipeline is outlined in Fig. 2a. Raw reads (fastq files) were first quality checked with FastQC. Filtered reads were aligned to the appropriate reference genome using BWA, and duplicate reads were marked using Picard. As the tumor and blood BAM files were merged to detect the somatic variants, the SNVs and indel calling were performed using two separate callers to assist in identifying true-positive somatic variant calls while reducing the false-positive calls.

WES metrics and summary statistics for each specimen are shown in Fig. 2b. We generated nearly 100 billion total reads from WES, for an average mapped coverage of 66–98. To evaluate the overall quality of the variant data, germline SNVs were called, and the transition to transversion and 135 dbSNP (Single Nucleotide Polymorphism Database) concordance ratios were calculated. We used the GATK and SAMtools programs to identify somatic SNVs and indels, respectively, and found a high validation rate for the variants called by program. The identified variants were combined into single file and annotated using ANNOVAR to add information, such the gene variants, consequence of the mutation (nonsynonymous, nonsense, etc.), and information from databases (e.g., PolyPhen2, SIFT, dbSNP, OMIN, and COSMIC). The final output of the pipeline consisted of a single file containing the annotated variants from the tumor and blood samples. These analyses indicated that no biases were encountered with respect to nucleotide substitutions. SNPs identified in the data were strongly correlated with common genetic variations, indicating that high-quality variant calling was performed.

### Novel germline mutation in the *MEN1* gene

The nine exons of the *MEN1* gene as well as their flanking introns were amplified and subjected to Sanger sequencing. Herein, we confirmed four germline variants of the *MEN1* gene by WES and Sanger sequencing, and the distribution is shown in Table 2. Mutations were found in exons 9 and 10 (one frameshift deletion, one missense mutation, and two synonymous variants). A novel germline mutation (c.1382_1383delAG/p.E462GfsX69) was newly confirmed in the present study, and the other three have been reported previously in the 1000 Genome Project and the Korean control exome dataset. This novel germline mutation (c.1382_1383delAG) was also present in all four tumor samples according to the WES analysis. The brother and son of the proband did not harbor this mutation (Fig. 1h). All variants were confirmed based on the NCBI SNP database (http://www.cnbi.nlm.nih.gov/SNP) and the Human Gene Mutation Database (http://www.hgmd.org).

**Table 2:** Germline mutation in the multiple endocrine neoplasia type1 (*MEN1)* gene identified by whole-exome sequencing and confirmed

Gene	Chr	Chr_start	Ref_base	Alt_base	Nucleotide change	Protein change	Mutation type	dbSNP135	1000G_2011 Oct_AF^a^	KRB_AF(622)^b^
*MEN1*	chr11	64,572,018	A	G	c.A1621G	p.T541A	nonsynonymous_SNV	rs2959656	0.85	0.75
***MEN1***	**chr11**	**64,572,256**	**AG**	**–**	**c.1382_1383delAG**	**p.E461GfsX69**	**frameshift_deletion**	**Novel**	**–**	**–**
*MEN1*	chr11	64,572,557	T	C	c.T1299C	p.H433H	synonymous_SNV	rs540012	0.98	1
*MEN1*	chr11	64,572,602	C	T	c.C1254T	p.D418D	synonymous_SNV	rs2071313	0.31	0.39

^a^Allele frequencies for each variant were estimated based on phase 1 genotypes from the 1000 Genomes project

^b^Korean control exome dataset, which includes exome data for 622 Koreans

### Detecting loss of heterozygosity

Oligonucleotide-based SNP genotyping array has been used to measure physical copy number and genetic aberrations, such as LOH. We included three additional MEN1 parathyroid specimens and one pancreatic specimen for which there was a paired germline sample (blood). Three tissue samples (left upper, left lower, and right upper parathyroid) had chromosomal allelic imbalance events (LOH with chromosomal loss).

Additional file 2: Figure S1 illustrates a greater degree of chromosomal allelic imbalance across the MEN1 tumor genomes compared with that in the blood sample. The SNP array collects intensity and allelic information, allowing two different genomic profiles to be generated. The log2 intensity ratio measures the intensity of a sample relative to a reference intensity, and the B allele frequency (BAF) measures the allele frequency at a particular SNP along the genome. The expected BAF value at a normal (non-LOH) polymorphic position is 0.5, and a significant deviation from 0.5 identifies LOH. LOH can easily be observed using the BAF plot by noting an absence of heterozygotes. Of note, there was consistent LOH of chromosome 11 (where the MEN1 gene resides) across all three of the parathyroid tumor samples from the patient with MEN1, whereas the pancreatic tumor exhibited no chromosomal LOH events

### Identifying tumor-specific somatic mutations

WES was performed on tumor and constitutional DNA samples from the patient with MEN1. The raw sequencing data were mapped to the hg19 reference human genome, the variants were identified, and tumor and constitutional blood sequences from each tumor were cross-referenced to identify somatic variants unique to each tumor genome.

WES was performed on the three parathyroid and one pancreatic tumors demonstrated an average of 180 variants in GATK and 389 in SAMtools. Additional file 1: Table S1 summarizes the number of somatic variants observed in the parathyroid and pancreatic tumors. Total somatic variants were examined using computational metrics and manual read alignment analysis to identify mutations with a high likelihood of being false positives.

The variants identified were annotated based on novelty, impact on the encoded protein, and conservation. Across all four tumor types, nine variants were identified in the coding region; two events were detected in the left upper parathyroid, three in the left lower parathyroid, two in the right upper parathyroid, and two in the pancreas. Of these events, we identified three indels and six nonsynonymous SNVs. A summary of the somatic variants identified in each tumor are shown in Table 3. Nine somatic variants, which were not present in the leukocyte samples, and, therefore, were not germline. Two somatic functional mutations outside the MEN1 gene were identified in the pancreatic insulinoma by WES. No somatic *MEN1* gene mutation was found in any of the four tumors.

**Table 3:** Somatic variants in different tumors by whole-exome sequencing

Gene symbol	Chr	Nucleotide change	Protein change	Variant type	Mutation type	dbSNP135	1000G_2011 Oct_AF	Variant allele frequency^a^				
								Parathyroid Left upper	Parathyroid Left lower	Parathyroid Right upper	Pancreas	Blood
*KIF21B*	chr01	c.G1738C	p.E580Q	SNV	nonsynonymous	–	–	**0.45**	0.00	0.00	0.00	0.00
*SRCAP*	chr16	c.C4012T	p.L1338F	SNV	nonsynonymous	–	–	**0.38**	0.00	0.00	0.00	0.00
*GRK6*	chr05	c.G241C	p.V81 L	SNV	nonsynonymous	–	–	0.00	**0.17**	0.00	0.00	0.00
*C9orf100*	chr09	c.G806C	p.S269 T	SNV	nonsynonymous	–	–	0.00	**0.33**	0.00	0.00	0.00
*CILP2*	chr19	c.C2173T	p.R725C	SNV	nonsynonymous	–	–	0.00	**0.50**	0.00	0.00	0.00
*CIR1*	chr02	c.724_725insGA	p.K242 fs	Indel	frameshift	–	–	0.00	–	**0.56**	0.00	0.00
*CNGA1*	chr04	c.316_317insG	p.K106 fs	Indel	frameshift	–	–	0.00	0.00	**0.83**	0.00	0.00
*ZNF429*	chr19	c.A1978T	p.R660X	SNV	stopgain	–	–	0.00	0.00	0.00	**0.21**	0.00
*SCARF2*	chr22	c.2239_2240insG	p.P747fs	Indel	frameshift	rs5844420	–	0.00	0.00	–	**0.40**	0.00

^a^Variant allele frequency observed in exome sequencing

## Discussion and conclusions

Genome-wide investigation of tumor and blood samples using WES and SNP array detected a novel MEN1 germline mutation (first hit) and tumor somatic mutations (second hit), confirming the two-hit model for tumor-suppressor genes. The heterogeneity between tumors (LOH in parathyroid tumors and no LOH in the pancreatic tumor) within a single patient suggests polyclonal tumorigenesis related to a predisposition arising from a single germline mutation.

The novel MEN1 germline mutation (c.1382_1383delAG; p.E462GfsX69) discovered in the present study was discovered by WES and confirmed by Sanger sequencing. This mutation was found in all four tumor samples and the blood sample and has not been reported in dbSNP135, the 1000 Genome Project, or the Korean control exome dataset. The brother and son of the proband did not harbor this mutation. The present study contributes to medical science by reporting a novel MEN1 germline mutation causing MEN1 syndrome and supports the clinical applicability of using WES to discover a causative mutation in the proband and to perform genetic testing on family members.

**Fig. 3:**
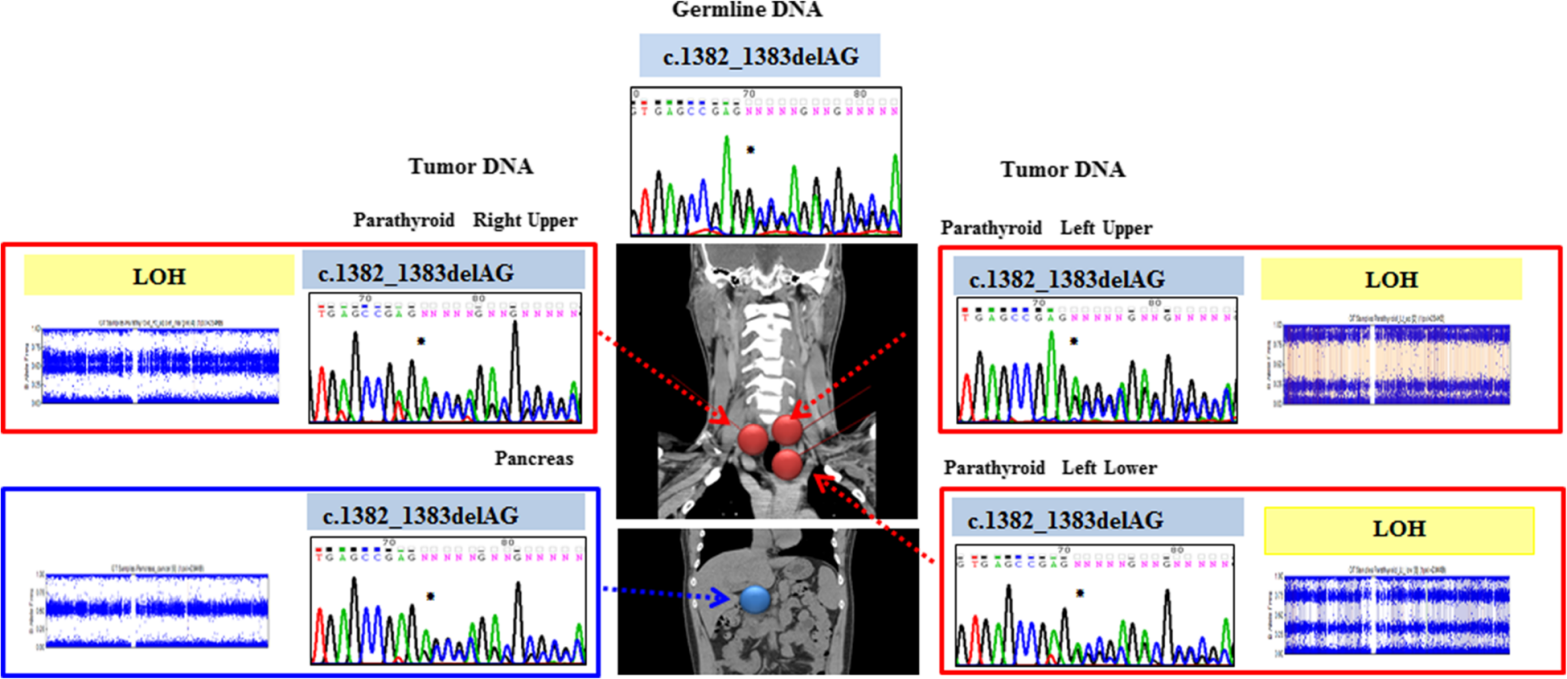
Analysis of multiple endocrine tumors from the same patient. Computed tomography scans and the various nodules present on both the parathyroid glands (red arrow) and pancreas (blue arrow) of the same patient are shown. Each nodule shows the germline defect (blue). A second alteration (yellow) differed between the parathyroid gland and pancreas

The LOH analysis detected the presence of chromosome 11 (where the MEN1 gene resides) LOH with chromosomal loss in all three parathyroid tumors. These findings demonstrate that the MEN1 germline mutation (first hit) and the chromosome 11 LOH (second hit) caused biallelic inactivation of the tumor suppressor MEN1 gene, which drives tumorigenesis of these tumors (Fig. 3). Additional chromosomal abnormalities were detected in the left lower part of the parathyroid tumor, suggesting the occurrence of possible genome instability and passenger mutations, as reported previously in similar studies on parathyroid tumors [14, 15]. No sign of LOH was found in the pancreatic tumor sample, suggesting that the mechanism of a MEN1 pancreatic tumor may be different from that of a MEN1 parathyroid tumor. These findings suggest that polyclonal tumorigenesis is related to a predisposition arising from a single germline mutation in MEN1 syndrome.

The somatic mutation analysis using WES revealed several possible driver mutations (other than MEN1). Because the pancreatic tumor did not harbor the MEN1 inactivation mutation (e.g., chromosome 11 LOH in the parathyroid tumors), we tried to determine the possible driver mutations involved in tumorigenesis in addition to the haploinsufficiency of the germline MEN1 mutation. One stop-gain mutation in the *ZNF429* gene and one frameshift mutation in the S*CARF2* gene were discovered in the pancreatic tumor sample. These mutations may make a contribution to the tumorigenesis that is in addition to that made by the predisposition arising from the germline MEN1 mutation. However, these somatic mutations from the parathyroid and pancreatic tumors may simply be passenger mutations resulting from tumorigenesis. It is difficult to distinguish a driver mutation from a passenger mutation in tumor samples using the WES approach.

We discussed a WES base analytical pipeline that detected germline and somatic variations by performing variant calling using two algorithms (GATK and SAMtools) to assist in identifying true-positive variant calls. To increase the validation rate, SNVs and indels were filtered with a mapping quality score > 58 in GATK and SAMtools. According to the SNP quality (SNPQ) and strand bias (SB) analyses, the variants were filtered with SNPQ ≥300, SB < −10 in GATK and SNPQ ≥40, and 20 ≤ AF% < 80 in SAMtools. These parameters are based on a previous study [16].

Here, we compared the WES platform results, which use depth of coverage and alternative allele frequencies from mapped sequenced reads to estimate LOH, with those of the SNP genotyping array. In the WES analysis, homozygous alleles were observed in chromosome 11 in all three parathyroid tumor samples, indicating LOH. Array Comparative Genomic Hybridization (aCGH) and SNP genotyping arrays have been widely used as standard methods to detect copy-number variation and LOH. However, with the rapid increase in genomic and exomic sequencing, LOH can be detected by WES, extending the ability of this powerful approach [17]. The resolution of these methods is limited by depth of coverage and the capture probe design. These data suggest that a WES-based platform is sufficient for identifying the genetic causes of various diseases in a diagnostic analysis and can be used to estimate germline and somatic mutations, as well as LOH simultaneously.

NGS allows characterization of genetic diseases with high accuracy and low cost. In particular, WES is an alternative approach to discovering variants that offers high specificity and sensitivity [18] and can be used to identify causative mutations in a number of Mendelian disorders, including monogenic diseases, as most causative mutations are on protein-coding regions. It also has the potential to identify de novo genetic variations through the WES platform. For these reasons, WES could be a surrogate to discover medically relevant genomic variations and diagnose genetic diseases at a genome-wide scale.

We identified a novel MEN1 germline mutation and tumor-specific somatic variants in a patient with MEN1 and multiple parathyroid and pancreatic tumors. Heterogeneity between tumors within a single patient suggests polyclonal tumorigenesis related to a predisposition arising from a single germline mutation. The present study demonstrates the clinical applicability of the WES strategy, which uses multiple tumor samples and a blood sample, to investigations of disease pathogenesis and evaluations of clinical genetics for patients and family members.

## Additional files

Additional file 1: Table S1. Number of somatic variants observed in four tumor types. (DOC 64 kb)

Additional file 2: Figure S1. Analysis of loss of heterozygosity (LOH) by single-nucleotide polymorphism (SNP) arrays. (A) Chromosomal alteration (gain, loss, or loss of heterozygosity) in parathyroid and pancreatic tumors. LOH of 11 was detected in the parathyroid tumor. LOH can easily be observed using a B allele frequency (BAF) plot by noting the absence of heterozygotes. (B) Chromosome 11 from parathyroid and pancreas. The upper part of the panel shows genotypes of the SNPs expressed as the BAF. The lower part of the panel shows the DNA copy number expressed on a base-2 log scale (log ratio), and the red line corresponds to two copies of DNA. (TIFF 789 kb)

## Abbreviations

aCGH: Array comparative genomic hybridization
AF: Allele frequency
BAF: B allele frequency
CT: Computed tomography
GATK: Genome Analysis Toolkit
KRG: Korean Reference Genome
LogR: Log2 intensity ratio
LOH: Loss of heterozygosity
MEN1: Multiple endocrine neoplasia type 1
NGS: Next-generation sequencing
SAMtools: Sequence Alignment Map tools
SB: Strand bias
SIFT: Sorting Intolerant Form Tolerant
SNPQ: Single nucleotide polymorphism quality
SNVs: Single nucleotide variants
WES: Whole-exome sequencing
