# Supplementary material for: Genetic analysis of parathyroid and pancreatic tumors in a patient with multiple endocrine neoplasia type 1 using whole-exome sequencing
Source: BMC Med Genet. 2017 Oct 2;18:106. doi: 10.1186/s12881-017-0465-9 (PMC5625714; doi:10.1186/s12881-017-0465-9)
Supplement: Supplementary file 1 — Number of somatic variants observed in four tumor types. (DOC 64 kb) [file 12881_2017_465_MOESM1_ESM.doc]

**Table S1. Number of somatic variants observed in four tumor types.**

| **Parameter** | **Parathyroid  Left upper** | **Parathyroid  Left lower** | **Parathyroid  Right upper** | **Pancreas** |
| --- | --- | --- | --- | --- |
| **GATK** | 188 | 183 | 158 | 194 |
| Nonsynonymous_SNV | 178 | 176 | 150 | 184 |
| Stop-gain_SNV | 5 | 3 | 4 | 5 |
| Frameshift_indel | 4 | 3 | 3 | 4 |
| Inframeshift_indel | 1 | 1 | 1 | 1 |
| **SAMtools** | 432 | 222 | 659 | 246 |
| Nonsynonymous_SNV | 367 | 166 | 532 | 182 |
| Stop-gain_SNV | 29 | 16 | 55 | 13 |
| Frameshift_indel | 14 | 13 | 49 | 17 |
| Inframeshift_indel | 22 | 27 | 23 | 34 |
| **GATK and SAMtools intersection** | 2 | 3 | 2 | 2 |
| Nonsynonymous_SNV | 2 | 3 | 0 | 0 |
| Stop-gain_SNV | 0 | 0 | 0 | 1 |
| Frameshift_indel | 0 | 0 | 2 | 1 |

SNV, single nucleotide variant
