# Supplementary figures and images for: Genetic analysis of parathyroid and pancreatic tumors in a patient with multiple endocrine neoplasia type 1 using whole-exome sequencing
Source: BMC Med Genet. 2017 Oct 2;18:106. doi: 10.1186/s12881-017-0465-9 (PMC5625714; doi:10.1186/s12881-017-0465-9)

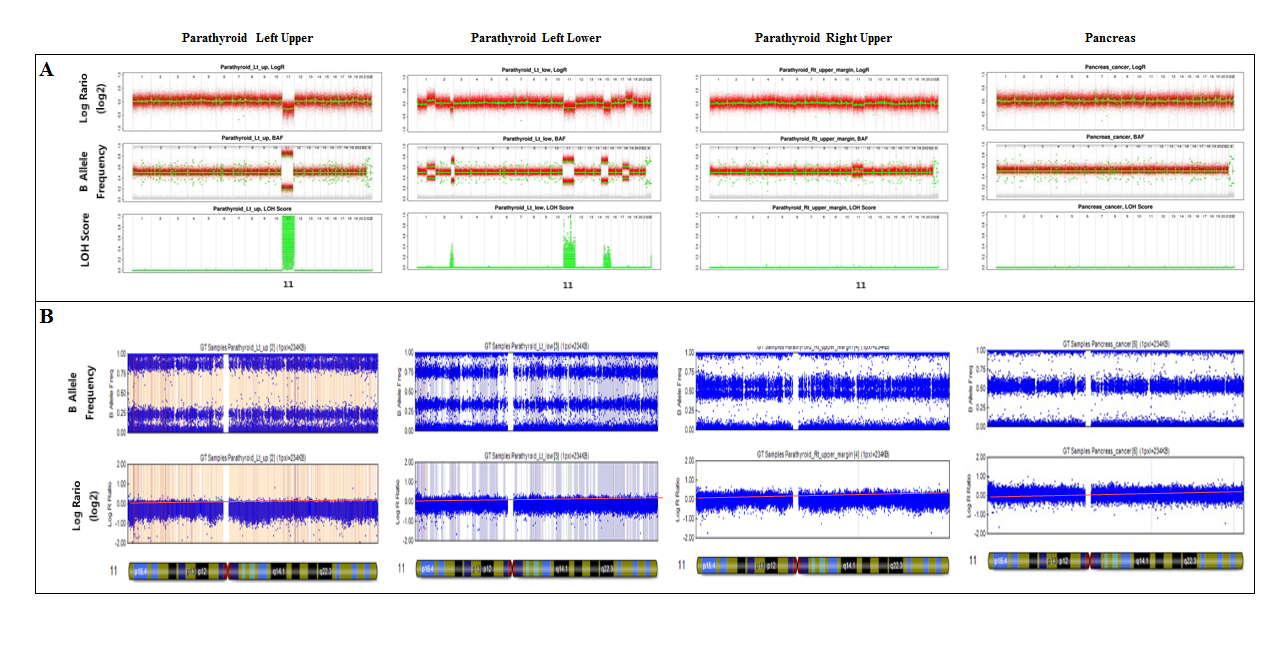

Supplement: Supplementary file 2 — Analysis of loss of heterozygosity (LOH) by single-nucleotide polymorphism (SNP) arrays. (A) Chromosomal alteration (gain, loss, or loss of heterozygosity) in parathyroid and pancreatic tumors. LOH of 11 was detected in the parathyroid tumor. LOH can easily be observed using a B allele frequency (BAF) plot by noting the absence of heterozygotes. (B) Chromosome 11 from parathyroid and pancreas. The upper part of the panel shows genotypes of the SNPs expressed as the BAF. The lower part of the panel shows the DNA copy number expressed on a base-2 log scale (log ratio), and the red line corresponds to two copies of DNA. (TIFF 789 kb) [file 12881_2017_465_MOESM2_ESM.tif]
